# Ringworm and Alopecia Areata

**Published:** 1898-09

**Authors:** 


**Ringworm and Alopecia Areata.** By H. Aldersmith, M.B. Fourth Edition. Pp. xvi., 327. London: H. K. Lewis. 1897.

This fourth edition is about four times the bulk of the last, which was issued in 1885. We note that treatment of the various kinds of ringworm occupies 141 pages of a work containing

313 pages. That so much should be required for the details of cure argues badly for its efficiency, but this difficulty is not the author's fault. He certainly is one who has laboured hard and carefully to improve the treatment of these very troublesome complaints, but we shall not feel content until the various plans suggested can be brought into the compass of say twelve pages.

The plurality of fungi causing ringworm are well considered, and M. Sabouraud's views on this subject are well stated and carefully criticised. Plates are often very useful, and we have very little fault to find with the seven which are delineated in this work and which chiefly illustrate the different fungi; but the lack of indicating figures in plates i., ii., and iii. is to be regretted, and also as to how the order of sequence must be understood, for in plate i. the drawings go from below upwards, whilst in plates ii. and iii. they evidently count from above downwards; plate ii., besides, is a sad reflection upon the efficacy of treatment, for the illustrations display the virility of a "stump" in a case which had been under care for seven years!

Modes of cure other than the author's are not quite fairly dealt with. In carrying these out, at least the prescribed and proper precautions should be taken. Thus, if a nurse left a caustic solution too long upon the head, as narrated on page 189, either she had been badly instructed or was herself very much to blame.

Dr. Aldersmith devotes nearly thirty pages to the description of his croton-oil treatment of obstinate ringworm, and it clearly is a very subtle remedy, far too much so for the busy and ordinary practitioner to take in hand, or even for a hospital specialist, unless he has a good staff of well-trained assistants; but what would the latter say if a nurse had rubbed the croton-oil into the diseased hairs instead of having it applied very carefully by means of a fine needle, specially constructed, and of which a very clear drawing is given?

The book is published in an attractive form, and is a valuable addition, in many respects, to what is really a too bulky literature on ringworm disease.

